# Socio-emotional readiness and classroom ecology in 5th-grade EFL classrooms: a responsive evaluation of the English curriculum in Türkiye

**DOI:** 10.3389/fpsyg.2026.1741565

**Published:** 2026-02-06

**Authors:** Hateme Aysel Kuzu, Eray Egmır

**Affiliations:** Department of Curriculum and Instruction, Afyon Kocatepe University, Afyonkarahisar, Türkiye

**Keywords:** classroom ecology, communicative language learning, emotional labor, responsive curriculum evaluation, socio-emotional readiness, young EFL learners

## Abstract

**Introduction:**

This study examines the socio-emotional and interactional dynamics shaping young learners’ engagement with the 5th-grade English curriculum in public secondary school contexts in Türkiye. Drawing on a responsive curriculum evaluation approach and grounded in classroom ecology and socio-cultural learning perspectives, the study focuses on how socio-emotional readiness, instructional mediation, and contextual demands shape communicative language learning.

**Methods:**

A qualitative case study design was employed. Data sources included curriculum document and textbook analysis, classroom observations, semi-structured interviews with seven English teachers, one teacher focus group, and a separate focus group with seven 5th-grade students. Data were analyzed using inductive thematic analysis.

**Results:**

Findings indicate that students’ participation in spoken English activities was closely associated with emotional security, peer-related evaluative concerns, and a sense of classroom belonging. Teachers reported experiencing significant pressures related to curriculum pacing and assessment expectations, which constrained opportunities for communicative interaction and intensified emotional labor. The analysis also revealed context-bound misalignments between intended communicative outcomes and enacted classroom practices, shaped by limited instructional time and the emphasis of instructional materials on vocabulary recall rather than contextualized use.

**Discussion:**

The findings highlight the importance of attending to emotional safety, interactional support, equitable material conditions, and adaptive instructional pacing within specific classroom contexts. From a responsive evaluation perspective, the study demonstrates how socio-emotional and ecological constraints influencing curriculum enactment can be made visible beyond outcome-based indicators, offering implications for curriculum support, teacher development, and communicative language pedagogy.

## 1 Introduction

Curriculum evaluation approaches vary in the extent to which they emphasize learning outcomes, lived experiences, and contextual realities. While product-oriented evaluation prioritizes measurable achievements, participant-oriented models foreground the meanings, values, and experiences of learners and teachers who actively shape curriculum implementation (Fitzpatrick et al., 2004; Stufflebeam and Shinkfield, 1985). Within this tradition, Stake’s responsive evaluation model highlights the importance of attending to emergent classroom concerns, relational dynamics, and contextual variation (Stake, 1983; Stake, 2003). Rather than judging curricula solely on predefined objectives, responsive evaluation considers how curriculum intentions intersect with emotional, interactional, and ecological dimensions of classroom life (Greene and Abma, 2001; Endut, 2014).

This approach is particularly relevant in Türkiye, where revisions to the English language curriculum since 2004 have aimed to promote communicative competence, functional language use, and active learner participation (Tekin Özel, 2011; Demirtaş and Erdem, 2015). Later updates in 2012 and 2017 emphasized earlier exposure to foreign language instruction and greater attention to listening and speaking skills (Acar, 2019). However, documented challenges include limited instructional time, dense content sequencing, and learning environments that do not adequately support students’ socio-emotional readiness to engage in spoken communication (Merter et al., 2012).

The transition into 5th grade represents a sensitive developmental period characterized by identity negotiation, heightened peer awareness, and the need for emotional security (Coşkun, 2008). As students shift from primary-level teacher-centered structures to multi-teacher secondary settings, they encounter increased academic expectations and social comparison. These conditions influence their willingness to participate in communicative activities, while teachers simultaneously navigate pacing pressures, assessment expectations, and emotional labor in sustaining student engagement.

Therefore, evaluating the 5th-grade English curriculum requires an approach that recognizes curriculum as an experience shaped by socio-emotional readiness and classroom ecology. Stake’s responsive evaluation model is well suited to this task, as it foregrounds stakeholder perspectives and attends to how curriculum design interacts with relational and contextual conditions. This study employs the responsive evaluation model to examine how learners and teachers experience the implementation of the 5th-grade English curriculum in Türkiye. The evaluation focuses on four dimensions: (1) individual and environmental factors influencing engagement, (2) alignment of learning outcomes and assessment, (3) content density and sequencing, and (4) instructional conditions within the classroom.

## 2 Theoretical background

### 2.1 Socio-emotional readiness in young EFL learners

Socio-emotional readiness refers to learners’ emotional security, self-confidence, and perceived sense of belonging that enable them to participate in communicative classroom activities (Pekrun, 2017; Dewaele and Li, 2020). Young learners in the early adolescent period are particularly sensitive to peer evaluation and social comparison, which can heighten language learning anxiety and reduce willingness to speak in the target language (Coşkun, 2008; Boudreau et al., 2018). When students feel emotionally safe and supported, they are more likely to take linguistic risks, initiate interaction, and negotiate meaning (Dörnyei and Ushioda, 2011). Conversely, fear of negative evaluation, performance pressure, and unclear expectations can constrain communicative participation.

The transition to 5th grade in Türkiye coincides with developmental changes such as identity exploration and heightened awareness of peer judgment. These socio-emotional conditions shape how learners respond to classroom tasks and how they interpret success and failure in foreign language learning. Thus, socio-emotional readiness is not a peripheral factor but a prerequisite for communicative competence in young EFL learners.

### 2.2 Classroom ecology and interactional learning environments

Classroom ecology conceptualizes the classroom as a dynamic interactional system shaped by the relationships among learners, teachers, tasks, tools, and material settings (Doyle, 2006; van Lier, 2004). From this perspective, language learning emerges not solely from instructional input but from the affordances and constraints of the learning environment. Interactional space, peer collaboration, teacher mediation, and the emotional climate collectively shape opportunities for meaning-making (Walsh, 2011).

In EFL classrooms, the quality of ecological conditions strongly influences students’ communicative engagement. When instructional pacing is rigid, curriculum content is dense, or classroom routines prioritize accuracy over meaning-making, learners’ opportunities to use language authentically may be reduced. Moreover, teachers often experience emotional labor in managing students’ participation and maintaining a supportive climate, which can either facilitate or hinder communicative interaction.

### 2.3 Responsive curriculum evaluation

Participant-oriented curriculum evaluation approaches recognize that curriculum effectiveness cannot be captured solely through prescribed objectives or test-based outcomes (Fitzpatrick et al., 2004; Stufflebeam and Shinkfield, 1985). Stake’s responsive evaluation model foregrounds stakeholder experiences and focuses on how curriculum goals interact with classroom realities (Stake, 1983; Stake, 2003). By attending to learners’ and teachers’ perspectives, responsive evaluation acknowledges intended and unintended outcomes, contextual variation, and emotional and relational dimensions of curriculum enactment (Greene and Abma, 2001; Abma, 2006).

This approach is particularly appropriate for the 5th-grade English curriculum in Türkiye, which is grounded in constructivist principles emphasizing learner participation, communicative use of language, and active engagement (Tekin Özel, 2011; Demirtaş and Erdem, 2015; Acar, 2019). However, previous research indicates challenges such as limited instructional time, dense unit structures, and learners’ socio-emotional barriers to speaking in English (Merter et al., 2012). Therefore, responsive evaluation provides a suitable framework for examining how curriculum intentions align with classroom ecological conditions and learners’ socio-emotional readiness.

Drawing on socio-emotional learning perspectives, classroom ecology, and Stake’s responsive evaluation framework, this study focuses on how the 5th-grade English curriculum is enacted and experienced within everyday classroom contexts. Rather than approaching curriculum implementation through predefined effectiveness indicators, the study conceptualizes implementation as a lived, context-bound process shaped by students’ socio-emotional readiness, teachers’ instructional mediation, classroom ecological conditions, and the affordances and constraints of learning materials.

Recent post-2020 research has increasingly emphasized the socio-emotional and interactional dimensions of communicative language learning, particularly in relation to learners’ willingness to communicate, emotional safety, and classroom participation. Studies conducted in diverse EFL contexts suggest that communicative engagement is shaped not only by task design but also by peer dynamics, perceived evaluative risk, and affective classroom climate (Dewaele and Li, 2020; MacIntyre et al., 2021; Oga-Baldwin and Hirosawa, 2022). In parallel, curriculum enactment research highlights how performance-oriented accountability pressures and assessment regimes may constrain communicative intentions at the classroom level, leading to tensions between policy discourse and pedagogical practice (Priestley et al., 2015; Sato and Loewen, 2022). Drawing on this emerging body of work, the present study situates the evaluation of the Grade 5 English curriculum within contemporary discussions of socio-emotional readiness, classroom ecology, and context-sensitive curriculum implementation.

## 3 Method

### 3.1 Research design

This study employed a qualitative case study design informed by Stake’s responsive evaluation framework. The design aimed to examine how the 5th-grade English curriculum was enacted in real classroom settings, with particular attention to socio-emotional readiness, classroom ecology, and stakeholder perspectives. Responsive evaluation was adopted to foreground issues that emerged during implementation rather than to judge curriculum effectiveness against predetermined objectives. In this way, the design supported a context-sensitive examination of how curriculum intentions interacted with relational, emotional, and material dimensions of classroom practice.

#### 3.1.1 Responsive evaluation procedures

Consistent with Stake’s (1976) responsive evaluation framework, the evaluation process was implemented iteratively rather than as a linear sequence. Initial stages focused on defining the scope of the Grade 5 English curriculum through detailed document and textbook analysis to establish contextual familiarity.

Early individual interviews with teachers surfaced recurrent concerns related to instructional pacing pressures, students’ reluctance to participate in spoken English activities, and perceived limitations of instructional materials. In response to these emergent issues, subsequent data collection was adjusted to probe these concerns more explicitly. Classroom observations foregrounded interactional pacing, moments of student hesitation or withdrawal during oral activities, and the use of instructional materials to manage lesson flow. Likewise, follow-up focus group protocols with teachers and students were refined to explore socio-emotional dimensions of participation, perceptions of communicative risk, and experiences of curriculum enactment under performance-oriented conditions.

Stakeholder concerns were thus explored not only as reported perceptions but as evolving analytic focal points shaping observation and interview emphasis. This iterative movement between data collection and analysis allowed the evaluation focus to evolve responsively in line with stakeholder-identified issues, rather than being anchored to predefined effectiveness indicators.

Analytic interpretations were revisited through expert consultation, limited participant confirmation, and triangulation across documents, interviews, focus groups, and observations. These processes supported interpretive rigor by enabling critical dialogue, plausibility checking, and alignment between emerging themes and the evidential base.

#### 3.1.2 Research questions

Consistent with the principles of responsive evaluation, the analytic focus of this study was shaped around stakeholder-identified concerns that emerged during curriculum implementation. These concerns centered on students’ emotional comfort in communicative participation, teachers’ mediation under instructional constraints, and the alignment between curricular intentions and classroom realities.

Based on this analytic focus, the study addressed the following research questions:

*RQ1*: How do socio-emotional readiness factors (e.g., emotional security, peer-related concerns, sense of belonging) shape 5th-grade students’ participation in communicative English activities?

*RQ2*: How do teachers perceive and mediate the alignment between the intended communicative outcomes of the 5th-grade English curriculum and actual classroom practices?

*RQ3*: How do classroom ecological conditions (instructional pacing, interactional space, and material use) enable or constrain communicative language learning in 5th-grade EFL classrooms?

*RQ4*: From a responsive evaluation perspective, what tensions and priorities emerge among stakeholders (teachers and students) regarding the implementation of the 5th-grade English curriculum?

### 3.2 Case definition and context

The case in this study was defined as the implementation of the national 5th-grade English curriculum in public secondary school classrooms in Türkiye, examined through the perspectives of teachers and students and through direct classroom observation. The case was bounded by (a) a single grade level (5th grade), (b) a common national curriculum and textbook and (c) routine instructional conditions within public schools.

The study was conducted during the 2023–2024 academic year in public secondary schools located in Afyonkarahisar, a mid-sized province that reflects typical curriculum implementation conditions in Türkiye. Classroom observations were conducted in two public secondary schools with contrasting ecological characteristics: School H and School A.

School H is located in the district center and serves students from multiple surrounding neighborhoods. As a result, the student population reflects considerable diversity in residential background. Class sizes typically range from 15 to 20 students. The school has relatively strong physical infrastructure, with multiple sections at each grade level and widespread use of interactive whiteboards. Students attend the school both on foot and through transportation services. Based on district-level assessment results, the school is characterized as demonstrating mid-range academic performance within the district.

School A is situated within the premises of an industrial facility and was originally established to serve the children of factory employees. Over time, it has also begun enrolling students from outside the immediate facility area. The school is smaller in size, with typically one section per grade level and average class sizes ranging from 25 to 30 students. The student population predominantly reflects professional occupational backgrounds. According to information provided by district education authorities, School A is consistently among the highest-performing schools in district-level academic assessments.

A noteworthy contrast was observed between School H and School A. While School H, with its larger and more diverse student body, displayed more visible fluctuations in participation and emotional readiness, School A tended to exhibit more stable engagement patterns but stronger exam-orientation pressures. These contextual differences shaped how teachers navigated pacing, interaction, and support, reinforcing the ecological nature of curriculum enactment.

**Figure 1:**
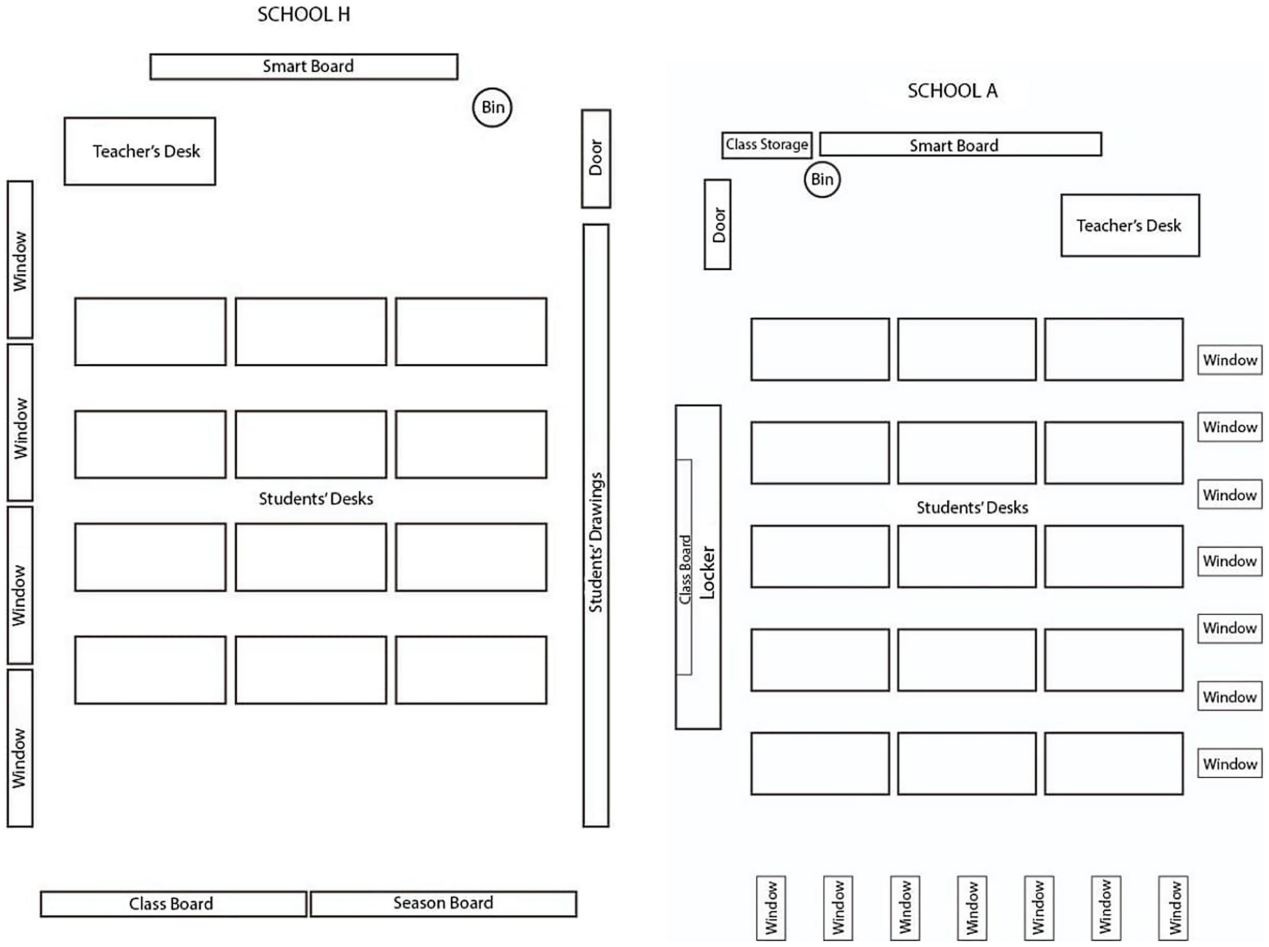
Sketches of the observed classrooms.

These two schools were selected to allow examination of how the same national English curriculum is enacted under differing classroom ecologies, particularly in terms of student composition, instructional pacing, and participation dynamics (see Figure 1).

### 3.3 Sampling strategy and participants

#### 3.3.1 School selection

Typical case sampling was used to select schools that reflected standard curriculum implementation conditions rather than extreme or exemplary cases. Schools were considered “typical” based on the following criteria:

- implementation of the national English curriculum without local adaptation,
- average class size and instructional hours consistent with national regulations,
- absence of selective admission procedures, and
- stable staffing patterns without recent structural reforms.

Typical case sampling is a purposive sampling strategy commonly used in qualitative research to examine phenomena as they occur under ordinary, non-extreme conditions, allowing researchers to capture patterns that are broadly representative of everyday practice (Patton, 2015; Creswell and Poth, 2018). In the context of curriculum evaluation, this approach is particularly appropriate when the analytic aim is to understand how policy intentions are enacted in routine classroom settings rather than to document best practices or problem-saturated cases (Stake, 2006). By focusing on schools that operate under standard structural and instructional conditions, the study sought to illuminate context-bound dynamics of curriculum implementation that are likely to be encountered in similar public school contexts. This sampling strategy was chosen to enhance the transferability of findings to comparable public school contexts.

#### 3.3.2 Teacher participants

A total of seven English teachers participated in the study (*N* = 8). Individual interviews were conducted with seven teachers (n_individual interview_ = 7). The teacher focus group included eight participants: the same seven interviewees plus one additional teacher who did not take part in the individual interviews but participated only in the focus group (n_focus group_ = 8). All teachers were graduates of Faculties of Education and had between six and 9 years of professional teaching experience. Teachers were actively teaching 5th-grade English during the data collection period. Table 1 presents the demographic characteristics of the participating teachers.

**Table 1:** Demographic characteristics of teachers.

Teacher code	Gender	Graduation program	Experience
Teacher E.	Female	Faculty of Education	6 years
Teacher M.	Female	Faculty of Education	9 years
Teacher B.	Female	Faculty of Education	7 years
Teacher S.	Male	Faculty of Education	8 years
Teacher Ç.	Male	Faculty of Education	9 years
Teacher V.	Male	Faculty of Education	8 years
Teacher A.	Male	Faculty of Education	8 years
*Teacher C.	Male	Faculty of Education	7 years

*Participated only in the focus group interview.

#### 3.3.3 Student participants

Student perspectives were collected through a focus group interview with seven 5th-grade students (three female, four male), aged 10–11 years. Students were recruited from School A, one of the observed schools, on a voluntary basis. Teachers informed students about the study and distributed invitations, but did not nominate or select participants based on academic achievement, behavior, or perceived proficiency. Participation was limited to students who expressed willingness to take part and whose parents provided written consent.

The focus group was designed to explore students’ experiences of classroom participation, emotional comfort, and engagement in English lessons rather than to assess individual language proficiency. No formal proficiency testing was administered. Students’ prior exposure to English reflected the standard primary-school sequence of the national curriculum.

### 3.4 Data collection

Data were collected from multiple qualitative sources to support triangulation and analytic depth.

#### 3.4.1 Document analysis

Document analysis was conducted to support in-depth examination of curriculum intentions and instructional conditions. The official Grade 5 English curriculum issued by the Ministry of National Education was analyzed in terms of stated objectives, content structure, sequencing, and communicative emphasis. To ensure content familiarity and contextual coherence, the nationally prescribed Grade 5 English textbook approved by the Board of Education was also examined.

In addition to official documents, supplementary instructional materials used in the participating schools were identified during interviews and observations with parental consent. Accordingly, commercially published supplementary resources employed by different schools were included in the analysis. Across all documents, analytic attention focused on content variety, presentation formats, quantity of activities, visual–textual balance, and the extent to which materials supported communicative language use. Findings from document analysis were reported through detailed descriptive summaries and compared with interview and observation data to support triangulation.

Document analysis focused on how communicative outcomes articulated in the curriculum were operationalized in the textbook through task types, sequencing, and interactional demands. Particular attention was paid to whether speaking and listening outcomes were supported through open-ended, interactional tasks or primarily through closed-format activities such as vocabulary matching, sentence completion, and recognition-based exercises. Units were examined comparatively to identify recurring patterns rather than isolated instances.

#### 3.4.2 Interviews and focus group procedures

Semi-structured individual interviews were conducted with seven English teachers from two public secondary schools. Interviews were carried out face-to-face in Turkish to allow nuanced expression and lasted between approximately 35 and 55 min.

An initial interview protocol consisting of 12 open-ended questions was developed in line with the study’s aims and research questions. The protocol was reviewed by two experts in Curriculum and Instruction (one associate professor and one assistant professor) and revised accordingly. A pilot interview was then conducted with a Grade 5 English teacher outside the study group to identify strengths and weaknesses of the protocol. Following expert feedback and pilot refinement, the final interview form was approved and used in data collection.

Based on preliminary analysis of individual interviews and classroom observations, a teacher focus group was conducted to enable deeper exploration of emerging themes. A semi-structured focus group protocol was developed using codes and themes identified during earlier analysis stages.

The initial focus group form included seven questions with supporting probes and was reviewed by an expert in Curriculum and Instruction (one associate professor). Following expert feedback, the protocol was refined to five core questions, each supported by two to three probes. After final approval and a teacher review outside the study group, the focus group was conducted with eight English teachers. The session lasted 106 min and was held in a neutral, easily accessible community space arranged to support group interaction.

To incorporate student perspectives, a semi-structured focus group interview was conducted with seven Grade 5 students. The protocol was developed based on the study’s research questions and findings from teacher interviews. An initial version included seven core questions with probes and was refined to five questions following expert review and a pilot application with a student group outside the study sample.

The student focus group was conducted in Turkish, lasted 95 min, and took place in a familiar home setting to support comfort and open participation. Questions focused on students’ experiences of classroom participation, emotional comfort, and engagement in English lessons.

#### 3.4.3 Classroom observations

Classroom observations were conducted as non-participant observations to document instructional pacing, interactional patterns, and the emotional climate of communicative participation. The researcher adopted an observer-as-outsider role and did not intervene in instructional activities. The classroom observations were designed to produce thick qualitative descriptions of interactional ecology rather than to generate quantifiable behavioral indicators. Accordingly, no standardized socio-emotional scales or structured participation mapping tools were applied. Instead, observations focused on interactional dynamics, emotional climate, participation patterns, and teacher mediation as they unfolded in real classroom contexts.

Observations were carried out in two public secondary schools selected to reflect contrasting classroom ecologies. At School H, three separate observation sessions were conducted across different days, covering a total of four consecutive English lessons (approximately 120 min). These lessons focused on routine curriculum units and allowed observation of instructional pacing under time pressure. At School A, four observation sessions were conducted across different days, covering a total of 160 min of instruction. Observations at this site included lessons in which teachers made explicit decisions to abbreviate or omit curriculum units due to pacing constraints.

All observed lessons followed the nationally prescribed Grade 5 English curriculum and textbook. Observations focused on lesson structure (introduction, development, closure), opportunities for spoken interaction, teacher mediation strategies, student participation patterns, and affective indicators such as hesitation, withdrawal, and peer responses.

To reduce reactivity, observations were conducted after an initial familiarization period during which the researcher was introduced to the class and remained present without recording data. During formal observation sessions, a structured observation form was used, supplemented by detailed field notes taken during and immediately after each lesson.

### 3.5 Data analysis

Data analysis followed an inductive thematic analysis approach. All interview and focus group transcripts, classroom observation notes, and document analysis records were analyzed by the researcher. The primary unit of analysis consisted of meaning units, defined as segments of text (phrases, sentences, or short exchanges) that conveyed a single idea related to participation, emotional climate, instructional mediation, or curriculum conditions.

An initial round of open coding was conducted through close, line-by-line reading of the data. Codes were generated inductively and documented in a provisional code list that functioned as an evolving codebook. As analysis progressed, codes were continuously refined, merged, or differentiated through constant comparison across data sources.

Interview and focus group questions were designed to elicit participants’ experiences related to classroom participation, emotional comfort, instructional mediation, curriculum pacing, and material use. During analysis, responses were not treated as question-bound categories; instead, analytically related questions were clustered and synthesized into broader thematic patterns. This approach allowed the analysis to move beyond surface-level question–answer structures and to identify cross-cutting issues that emerged consistently across data sources. Consequently, the reported themes represent interpretive syntheses of multiple questions rather than direct reflections of individual interview prompts.

In the second analytic phase, related codes were grouped into higher-order categories based on conceptual similarity. These categories were further examined through analytic memo writing, which was used to capture emerging relationships, tensions, and patterns across teacher, student, and observational data. Memoing played a central role in tracing how initial codes (e.g., “hesitation to speak,” “fear of peer judgment,” “rushing activities”) coalesced into broader thematic constructs such as the emotional climate of participation and performance-oriented classroom ecology.

Theme development was finalized through iterative review of coded data excerpts, analytic memos, and peer debriefing with experts in Curriculum and Instruction. Discrepant interpretations were discussed until conceptual agreement was reached, and interpretive decisions were documented to maintain an audit trail.

No qualitative data analysis software was used; instead, coding and memoing were conducted manually using annotated transcripts and analytic matrices to support close engagement with the data.

Throughout the analytic process, preliminary codes and emerging themes were reviewed through peer debriefing with experts in Curriculum and Instruction. In addition, selected interview and focus group sessions were audio-recorded and, where applicable, video-recorded, allowing the researcher to revisit non-verbal cues (e.g., hesitation, laughter, silence) during analysis. These checks supported interpretive accuracy, particularly in relation to emotional and interactional dimensions of classroom participation.

In the findings tables, the notation “n” refers to the number of coded data segments in which a given code appeared across the dataset. These counts are provided for descriptive transparency only and are not intended to indicate participant-level prevalence or statistical significance.

### 3.6 Trustworthiness and reflexivity

Reflexivity was treated as an ongoing analytic practice throughout data collection and analysis rather than as a post-hoc declaration. The researcher maintained reflexive notes at three stages: (a) after classroom observations, focusing on interactional dynamics, researcher positioning, and potential expectancy effects; (b) following each interview and focus group session, documenting initial analytic impressions, moments of uncertainty, and perceived power dynamics; and (c) during the coding and theme development process, where assumptions about curriculum implementation and student readiness were explicitly questioned and recorded.

Given the researcher’s professional background as an English language educator, particular attention was paid to monitoring potential biases related to instructional norms, assessment expectations, and teacher–student interaction patterns. Reflexive notes were revisited during analysis to examine whether emerging interpretations reflected participants’ accounts or the researcher’s prior pedagogical assumptions.

Peer debriefing was conducted at multiple points in the analytic process with two external experts in Curriculum and Instruction (one associate professor and one assistant professor). These sessions focused on the clarity of code definitions, the boundaries between categories, and the plausibility of emerging themes. Feedback from these discussions resulted in the refinement of theme labels, the consolidation of overlapping codes, and the rewording of interpretive claims to ensure alignment with the evidential base.

A limited and informal form of participant confirmation was also conducted to assess the interpretive plausibility of emerging themes. Following the initial coding and theme development, three teacher participants were contacted through brief face-to-face follow-up conversations conducted within 2 to 3 weeks after the completion of data analysis. During these conversations, the researcher verbally summarized the preliminary themes and interpretive patterns and invited teachers to comment on whether these interpretations reasonably reflected their classroom experiences. These exchanges typically lasted 10–15 min per participant. No participants expressed disagreement with the thematic interpretations or indicated misrepresentation. This process functioned as an interpretive plausibility check rather than as a formal validation procedure.

### 3.7 Language, translation, and ethical considerations

All interviews, focus group discussions, and classroom interactions were conducted in Turkish, the participants’ native language, to enable nuanced expression and minimize linguistic constraint. Audio-recorded data were transcribed verbatim in Turkish prior to analysis.

For publication purposes, selected excerpts were translated into English by the researcher, who is proficient in both Turkish and English and has professional experience in English language education. To ensure semantic accuracy and preserve contextual meaning, translations prioritized meaning-based equivalence rather than literal word-for-word rendering. During the translation process, attention was paid to maintaining participants’ intended tone, emphasis, and pragmatic meaning, particularly in relation to expressions of emotion, hesitation, and evaluative stance. Translated excerpts were reviewed against the original Turkish transcripts during analysis to minimize meaning distortion.

Ethical approval for this study was granted by the Afyon Kocatepe University Social and Human Sciences Scientific Research and Publication Ethics Committee (Decision No: 2022/96). All procedures were conducted in accordance with institutional and national ethical standards. Participation was voluntary. Written informed consent was obtained from all participating teachers, and written parental consent and student assent were obtained for all student participants. Teachers did not participate in or observe the student interview process. All data were anonymized prior to analysis and reporting.

### 3.8 Data availability statement

Because the dataset includes interviews and focus group data with minors and school-based participants, the full transcripts, audio files, and contextually identifiable materials cannot be shared publicly for ethical and confidentiality reasons. However, de-identified analytic materials (e.g., coded extracts, the coding framework, and documentation of theme development) can be made available upon reasonable request to the corresponding author, subject to ethical approval and confidentiality protections.

## 4 Findings

The analysis of interview, observation, and document data revealed four main themes characterizing the implementation of the 5th-grade English curriculum: (1) performance-oriented classroom ecology and the emotional climate of participation, (2) teacher emotional labor and instructional mediation, (3) misalignment between intended learning outcomes and instructional conditions, and (4) material constraints shaping the interactional ecology of the classroom. Together, these themes illustrate how curriculum implementation, within the studied contexts, is shaped by the emotional dynamics of classroom participation, instructional and organizational constraints, and the pedagogical work required to bridge the gap between the curriculum’s communicative intentions and learners’ lived experiences.

### 4.1 Theme 1: performance-oriented classroom ecology and the emotional climate of participation

The implementation of the 5th-grade English curriculum unfolded within a performance-oriented classroom ecology shaped by parental expectations, administrative monitoring, and pacing pressures. Teachers described navigating tensions between the curriculum’s communicative aims and institutional emphases on measurable outcomes. This ecology influenced not only instructional decision-making but also the emotional climate in which students engaged with spoken English activities.

Student accounts further illustrated how classroom participation was shaped by peer-related evaluative pressure. During the student focus group, when one student stated that they enjoyed English lessons and actively participated, a peer immediately responded, “I’m sure you participate a lot,” followed by collective laughter. Several students described withholding participation for fear of embarrassment. As one remarked, “Even if I know the answer, I don’t want everyone to look at me if I say it wrong,” while another noted that mistakes tend to “stay in people’s minds.” A third student summarized this logic succinctly: “It’s safer to stay quiet than to be embarrassed.” These accounts suggest that participation was framed not only as a learning behavior but also as a socially visible performance, carrying emotional risk. Teacher and student accounts together provide contextual grounding for the thematic patterns summarized in Table 2.

**Table 2:** Codes related to the socio-pedagogical context of curriculum implementation.

Code	*n*	Related quote
Pressure from parents and administration	13	“There is clear pressure from families. When mock-exam averages are low or a unit lags, complaints arrive quickly.” *(Teacher Ç.)*
Anxiety about completing the curriculum	9	“When I struggle to finish the curriculum on time, I feel anxious; sometimes I rush or skip activities.” *(Teacher E.)*
Students’ insufficient socio-emotional readiness	7	“Students do not arrive in Grade 5 with sufficient readiness; we are pushed back toward teacher-centered instruction.” *(Teacher S.)*
Sufficiency of physical facilities	6	“Interactive whiteboards and e-books help us move through activities quickly and sustain engagement.” *(Teacher V.)*

Teachers consistently described an instructional climate in which communicative engagement sometimes became secondary to demonstrable coverage and progression. As one teacher explained, “Parents become upset about English scores. Even when I explain improvement, they only see the number; I once received complaints for being ‘one unit behind.” During the teacher focus group, such comments were accompanied by lowered voices, pauses, and brief affirmations. While no participant explicitly attributed this to administrative surveillance, these interactional cues appeared to reflect perceived sensitivity around accountability expectations, resonating with teachers’ earlier statements about monitoring.

Pacing anxiety emerged as a persistent constraint. Teachers reported compressing activities, reducing scaffolding, or skipping tasks to align lessons with school-level plans and diagnostic testing calendars. “I try to include games and interactive work, but everything must fit into a monthly calendar. That makes me anxious and leads to rushing.” (Teacher E.). Classroom observations were consistent with these accounts. In four consecutive lessons at School H, activity cycles were typically limited to approximately 4–5 min, with incomplete book tasks reassigned as homework. At School A, one teacher explicitly decided to omit Unit 10, explaining, “It wouldn’t fit anyway; better to finish nine properly.”

These pressures appeared to intersect with students’ socio-emotional readiness. Teachers described many learners entering Grade 5 without established communication routines or confidence, particularly during whole-class spoken tasks. “Students come in more used to rote learning; they hesitate to take risks, so we end up carrying the cognitive load.” (Teacher S.). Observed moments of prolonged silence after prompts, reluctance to come to the board, and reliance on whispered peer support were interpreted as indicators of emotional hesitation surrounding oral participation.

Material affordances sometimes helped sustain engagement but did not necessarily resolve these dynamics. Interactive whiteboards, e-books, and digital platforms were valued for maintaining lesson flow, yet they were often employed to accelerate progression rather than deepen communicative space. For example, at School H, when confusion arose during a numbers activity, the teacher shifted rapidly to a chorus-based repetition video to “reset the rhythm,” and gap-fill exercises were frequently used to confirm comprehension before moving to the next segment.

Overall, within the observed classrooms, curriculum implementation operated in a tension field: institutional performance expectations narrowed instructional flexibility; students’ socio-emotional readiness constrained uptake; and material tools primarily supported momentum rather than extended communicative interaction. The pattern aligns with ecological perspectives emphasizing that communicative learning is co-constructed through emotional safety, relational dynamics, and contextual affordances (van Lier, 2004; Walsh, 2011; Dewaele and Li, 2020).

### 4.2 Theme 2: teacher emotional labor and instructional mediation in classroom enactment

Classroom enactment of the 5th-grade English curriculum appeared to depend heavily on teachers’ continuous instructional mediation and emotional labor. Teachers worked to sustain participation, regulate classroom tempo, and maintain student affective engagement while navigating fluctuating levels of socio-emotional readiness and curriculum pacing demands. Although certain units afforded embodied and experiential learning activities—such as role-play, direction-giving, or project-based tasks—lesson flow was frequently constrained by limited task variety and institutional expectations to complete units rapidly.

Although Theme 2 was primarily informed by teacher accounts, student perspectives offered complementary insight into how instructional mediation was experienced in practice. During the focus group interview, students noted that lessons often progressed quickly, with limited time to pause or revisit spoken English activities. One student explained, “Sometimes we move to the next thing before I understand the first one,” while another remarked, “If you miss it once, there is no time to ask again.” Several students linked this pace to hesitation in oral participation, noting that “when the lesson goes fast, it’s harder to speak because you’re not sure anymore.” These perspectives suggest that efforts to manage time and curriculum coverage—often described by teachers as necessary instructional mediation—were sometimes experienced by students as emotionally and cognitively demanding classroom conditions. Taken together, teacher and student accounts provide contextual grounding for the thematic patterns summarized in Table 3.

**Table 3:** Codes related to classroom enactment and instructional mediation.

Code	*n*	Representative quote
Vocabulary- and grammar-centered instructional routines	15	“We end up prioritizing time expressions and patterns; the structures push everything else to the background.” *(Teacher A.)*
Teachers’ emotional and design labor to sustain engagement	12	“I prepare fun things, but the curriculum does not always accommodate them; it works only when students demonstrate sufficient socio-emotional readiness to engage.” *(Teacher V.)*
Reliance on supplementary/smartboard-based materials due to limited task variety in the state book	10	“The book is light on activities, so we must use digital and external materials to keep the lesson going.” *(Teacher V., focus group)*
Suitability of specific units for practical/experiential tasks	6	“In the *In My Town* unit we built shops and practiced route directions — go straight, turn right — the children really paid attention.” *(Teacher Ç.)*

Teachers frequently described curriculum implementation as a continuous process of “holding the lesson together,” particularly when students hesitated or withdrew from communicative participation. This mediation involved adjusting task difficulty, shifting between whole-class and individual formats, re-modeling responses, reformulating prompts, and using affective strategies to reduce perceived risk. Many teachers emphasized that sustaining engagement required both cognitive and emotional orchestration. As one teacher described, “Even if the activity is good, it only works when students feel emotionally ready to engage; otherwise I have to redesign the task while teaching.” (Teacher V.)

Some units seemed to inherently support experiential learning and expand communicative space. In ‘In My Town’, for example, classroom observations showed students constructing small paper shops, creating neighborhood maps, and practicing spatial language through role-play. These lessons were characterized by heightened attention, collaborative energy, and willingness to attempt spoken English—even where accuracy was limited. However, teachers emphasized that such momentum was fragile and dependent on instructional time, class cohesion, and learners’ prior exposure. As one noted, “The class moves slowly but meaningfully; if the basics are weak, it is difficult to sustain that pace.”

Despite valuing communicative and embodied instruction, teachers frequently reverted to vocabulary drills and sentence-level grammar routines. This shift did not appear to stem from pedagogical preference but emerged as a pragmatic response to pacing pressures and learner uncertainty. Observational field notes broadly aligned with this pattern: lessons often began with vocabulary copying or smartboard translation activities, moved to brief grammar clarification, and concluded with short-form gap-fill tasks. When signs of disengagement appeared, audiovisual or game-based supports were commonly introduced to restore attention. These transitions helped preserve rhythm but did not always expand opportunities for extended communicative interaction.

Material resources played a central role in shaping instructional mediation. While interactive whiteboards and supplementary materials allowed teachers to respond flexibly, access was uneven across students. In several observed lessons, some students engaged actively with projected materials while others copied passively or disengaged. Teachers acknowledged these disparities but perceived limited alternatives given pacing expectations and assessment demands. As one teacher explained, “We must keep the lesson moving; otherwise we fall behind, and the students feel like they are failing.”

Overall, instructional mediation appeared to involve far more than selecting activities. Teachers navigated a complex interplay of performance expectations, fluctuating socio-emotional readiness, and material limitations while working to maintain students’ sense of competence and emotional safety. Within the observed contexts, these conditions sometimes narrowed the space for extended speaking and listening practice, leaving the curriculum’s communicative aspirations heavily reliant on teachers’ ongoing emotional and pedagogical labor.

This finding aligns with research emphasizing that communicative engagement in language learning is shaped by teachers’ affective mediation, classroom relational dynamics, and the interactional affordances available during instruction (Walsh, 2011; Dörnyei and Ushioda, 2011; Dewaele and Li, 2020).

### 4.3 Document analysis: curriculum and textbook emphases

Document analysis of the official Grade 5 English curriculum and the nationally prescribed textbook revealed a consistent emphasis on vocabulary recognition and form-focused activities, with comparatively limited opportunities for contextualized language use and extended communicative production. Although curriculum documents explicitly articulate communicative learning outcomes across listening, speaking, reading, and writing, the instructional materials operationalize these outcomes primarily through item-based exercises.

Across units, textbook activities were predominantly structured around word matching, gap-filling, labeling, and short-answer recognition tasks. Speaking and listening outcomes were typically represented through brief, tightly scripted prompts (e.g., choral repetition or short response cues) rather than open-ended interactional tasks that require sustained language use. When communicative activities were included, they were often positioned toward the end of units and framed as time-dependent or optional, limiting their instructional centrality.

In contrast, vocabulary- and grammar-focused activities appeared earlier in lessons and were more densely distributed across units, signaling their priority within the instructional sequence. This pattern suggests that while communicative competence is emphasized at the level of curricular intention, the material design of the textbook foregrounds coverage, recognition, and measurability over interactional depth and contextualized language use.

These documentary patterns provide an important contextual layer for interpreting teacher and student accounts reported below. In particular, they help explain teachers’ reliance on vocabulary drills and test-aligned routines and illuminate how material conditions contribute to the misalignment between intended outcomes and enacted classroom practices.

### 4.4 Theme 3: misalignment between intended outcomes and learning conditions

Although the official curriculum distributes learning outcomes across listening, speaking, reading, and writing, classroom enactment and assessment practices within the observed settings appeared to be shaped strongly by curriculum pacing demands and high-stakes exam expectations. Teachers frequently reported adapting, reframing, or “updating” the curriculum to align with test-oriented performance indicators. In their accounts, this process often displaced opportunities for extended communicative interaction and narrowed instruction toward knowledge that could be measured quickly and efficiently.

Student perspectives pointed to a perceived disconnect between curriculum expectations and everyday classroom learning conditions. During the focus group interview, students described the English content as repetitive and predictable. One student remarked, “It’s always the same topics; we already studied these in primary school,” a statement that was met with nodding and verbal agreement from peers. Another student explained that lessons often felt “predictable,” adding, “we know what will come next, so it’s not exciting to speak.” These experiences suggest that, for some learners, familiarity and repetition reduced interest and willingness to participate, making lessons feel routine rather than communicative or meaningful.

Document analysis provided complementary evidence that supported these perceptions. Across multiple units, textbook activities relied heavily on vocabulary lists, sentence-level substitution patterns, and recognition-based exercises, with comparatively fewer tasks requiring open-ended oral production or sustained interaction. Although communicative outcomes are clearly articulated in curriculum documents, the sequencing and density of textbook content appeared to prioritize coverage and recall, which may have constrained opportunities for meaningful language use in practice. For example, in the Festivals unit, speaking and listening are listed as core learning outcomes; however, textbook activities predominantly consist of number recognition (e.g., 100–1000), vocabulary matching, and sentence completion tasks. Opportunities for oral production are largely limited to brief choral repetition or optional pair prompts, with no sustained interactional tasks required. Similar patterns were observed across other units, where communicative outcomes were articulated at the curriculum level but operationalized through recognition- and recall-oriented activities in the textbook. A similar pattern was observed in the My Daily Routine unit. While the curriculum specifies communicative outcomes related to describing daily activities and exchanging personal information through spoken interaction, the textbook primarily operationalizes these outcomes through vocabulary matching, sentence completion, and short substitution exercises (e.g., “I wake up at…”, “I go to school at…”). Speaking opportunities are typically limited to controlled repetition or optional pair prompts without extended interactional support. As in other units, communicative outcomes are articulated at the curriculum level but are translated into recognition- and recall-oriented tasks in the instructional materials.

Taken together, teacher and student accounts, supported by document analysis, provide contextual grounding for the thematic patterns summarized in Table 4.

**Table 4:** Codes related to outcome–practice–assessment misalignment.

Code	*n*	Representative quote
Practice not aligned with stated outcomes	13	“Because of the theory–practice conflict, we update the content for exam preparation.” *(Teacher A.)*
Assessment not aligned with stated outcomes	10	“The curriculum asks for speaking, listening, writing, but in reality we must go exam-oriented.” *(Teacher S., focus group)*
Lack of skill-based assessment	8	“If we cannot build the skills, we cannot evaluate them; the required assessment does not happen.” *(Teacher A., focus group)*

Teachers emphasized that, while the curriculum promotes communicative production, everyday enactment was influenced by exam pressures embedded in the secondary school pathway, particularly through mock examinations and perceived high school entrance exam preparation demands. “Writing tasks exist, but parents value test scores more than speaking or writing; we inevitably give more room to tests.” (Teacher Ç.). As a result, some communicative tasks appeared to be reduced to short-form or symbolic activities rather than opportunities for extended, supported language use.

Teachers also described what they perceived as a structural contradiction between curricular intentions and enactment realities. Although the curriculum encourages patterned functional language with less emphasis on grammatical meta-language, classroom practice often drifted toward form-focused, accuracy-based routines to secure measurable results. As Teacher S. expressed: “The curriculum wants us to be speech-oriented, but students are habituated to note-taking and memorization, so we reshape the patterns and work on substitutions.”

Classroom observations across the lessons conducted in both schools reflected similar patterns of narrowing. In units intended to target oral and listening outcomes (e.g., numbers 100–1,000 in Festivals), lessons were frequently re-centered on copying, spelling drills, and board-based recitation, with brief choral repetition appearing later in the sequence. Reading activities typically emphasized recognition through sentence-by-sentence translation and comprehension checks (true/false, completion), while teachers sometimes reported skipping or abbreviating speaking tasks to “keep the flow” and avoid falling behind school pacing calendars.

Assessment practices further reinforced these tendencies. Although the curriculum advocates four-skill evaluation, teachers reported relying predominantly on multiple-choice and item-based assessment formats, particularly under parental and administrative score monitoring. One teacher summarized this instructional situation: “Instead of allocating one hour to listening and one to speaking, we spend two hours on test solving.” (Teacher A., focus group).

Students, in turn, appeared to internalize this performance logic, as reflected in teacher reports such as: “Why do two English questions when one math question counts more?” (Student paraphrased via teacher, focus group). This orientation discouraged risk-taking and reinforced minimal participation in communicative tasks, particularly when correctness seemed to outweigh expressive experimentation.

In sum, this theme highlights a context-specific pattern, within the observed classrooms, of movement from communicative intentions toward test-aligned coverage, contributing to misalignments between stated outcomes and enacted instructional and assessment practices. These patterns appeared to be reproduced by pacing calendars, score-based accountability expectations, and instructional time pressures that favored quick measurable outputs over supported, scaffolded communicative engagement.

This finding is consistent with research suggesting that when institutional demands prioritize measurable achievement, communicative language goals may become constrained by recognition-oriented tasks, reducing opportunities for authentic language use (Walsh, 2011; Dörnyei and Ushioda, 2011; Boudreau et al., 2018).

### 4.5 Theme 4: limitations of learning materials and resource use

This theme captures how the limitations of instructional materials—particularly the state-issued MoNE textbook—shaped both the opportunities and constraints for communicative learning within the observed classrooms. Teachers consistently characterized the official textbook as insufficient in depth, variation, and communicative task design. This perception aligns with the document analysis, which showed that the state-issued textbook provides limited scaffolding for open-ended oral tasks, instead emphasizing short, closed-format activities that can be completed quickly and individually. As a result, instruction frequently relied on supplementary workbooks, commercial publisher resources, and smartboard-based digital activities. However, inconsistent student access to these supplementary materials appeared to be associated with differentiated patterns of participation and uneven engagement.

Student accounts further illuminated the limited role of learning materials in supporting meaningful engagement with English lessons. During the focus group interview, several students reported that they did not actively use the coursebook; some indicated that they were unsure of its whereabouts, while others stated that they had stopped bringing it to school altogether. When asked about their reasons, students described the textbook as “too simple,” “not interesting,” and “something we had already seen in primary school.” One student commented, “There is nothing to do with it in class anyway,” suggesting that the textbook was perceived as peripheral to actual classroom practice.

Taken together, these accounts suggest that the officially prescribed materials were often perceived as lacking relevance, challenge, and instructional value, reducing their usefulness both inside and outside the classroom. Rather than functioning as scaffolds for communicative participation, the materials appeared, in these settings, to be loosely connected to students’ learning practices and interests. Teacher accounts, student perspectives, and observational evidence provide contextual grounding for the thematic patterns summarized in Table 5.

**Table 5:** Codes related to limitations of learning materials and resource use.

Code	*n*	Related quote
Insufficient textbook content	21	“The activities are not enough and do not reinforce the topics. We constantly need extra sources.” *(Teacher B., focus group)*
Reliance on supplementary resources	14	“We are always using external books or digital platforms because the main book is not adequate.” *(Teacher C., focus group)*
Unequal student access to materials	11	“Not every student can buy the supplementary book, so some follow, others just watch.” *(Teacher M., focus group)*

Teachers described the MoNE textbook as “repetitive,” “activity-light,” and lacking in scaffolding for meaningful oral or interactive practice. This placed much of the responsibility for creating communicative opportunities on teachers themselves, who supplemented the textbook with worksheets, external workbooks, or digital platforms. Consequently, curriculum enactment became increasingly dependent on teacher initiative, with the quality of learning opportunities shaped by teachers’ resource capacity and familiarity with additional materials. Several teachers noted that this process also increased planning workload.

Classroom observations suggested that these material constraints translated, at times, into visible participation disparities. During lessons where supplementary workbooks or digital tasks were projected on the smartboard, students who owned the workbook tended to follow tasks more confidently, while others appeared more dependent on teacher guidance or peer support. As one student expressed during the focus group, “We are just watching again. We cannot do anything without the book.” Such conditions appeared to limit opportunities for oral production, peer interaction, and autonomous task engagement, particularly for students without access to supplementary resources.

Teachers acknowledged this inequity but emphasized structural constraints: supplementary materials require additional purchase, and there was no consistently available mechanism ensuring equal access in the observed settings. As one teacher summarized, “We want to do more speaking work, but when half the class does not have the book, the lesson collapses.”

Overall, within the observed classrooms, limitations of the textbook and inconsistent distribution of supplementary materials appeared to contribute to fragmented learning opportunities, uneven participation, and reduced space for communicative practice, despite the curriculum’s stated learner-centered orientation. In this sense, the material environment functioned not only as a logistical backdrop but as a structuring condition shaping interactional ecology, participation hierarchies, and opportunities for spoken English use. This pattern aligns with ecological perspectives emphasizing that material affordances and access conditions co-construct participation possibilities and meaning-making opportunities in classroom settings (van Lier, 2004; Walsh, 2011).

## 5 Discussion

This study examined how the 5th-grade English curriculum in Türkiye is enacted within public secondary school classroom contexts, focusing on the socio-emotional dynamics of participation and the interactional ecology of learning. The findings suggest that the curriculum’s communicative intentions appeared to be constrained within the studied contexts by performance-oriented classroom climates, uneven student socio-emotional readiness, limited material affordances, and assessment practices privileging measurable and test-aligned outcomes. Together, these conditions were associated with context-bound misalignments between intended outcomes and enacted instructional practices.

From an ecological perspective, classroom participation emerged as a relational and affective process rather than a purely instructional one. Learners’ willingness to speak appeared to depend not only on linguistic knowledge, but also on emotional security, peer relations, and perceived interactional risk. Consistent with socio-emotional theories of language learning (Dewaele and Li, 2020; Boudreau et al., 2018), students in this study often withdrew from oral interaction when classroom conditions signaled high evaluation stakes or when uncertainty about accuracy outweighed perceived safety. In this sense, communicative learning was supported not only through structured tasks, but also through relational scaffolding and the normalization of linguistic experimentation. This finding is consistent with recent post-2020 research indicating that learners’ willingness to communicate is strongly shaped by emotional safety, peer evaluation, and perceived interactional risk rather than linguistic competence alone (Dewaele and Li, 2020; MacIntyre et al., 2021; Oga-Baldwin and Hirosawa, 2022).

However, the performance-oriented classroom ecology observed in this study appeared to limit opportunities to cultivate such conditions. Teachers described experiencing pacing pressures shaped by parental expectations, administrative monitoring, and assessment demands, which in turn compressed instructional time and constrained the depth of communicative engagement. This finding aligns with previous research suggesting that accountability structures and curricular pacing may incentivize content coverage over meaning-focused interaction (Walsh, 2011; Dörnyei and Ushioda, 2011). Within the observed settings, teachers’ emotional labor appeared to function as an important mediating mechanism that sustained lesson flow and student participation when other ecological supports were perceived as limited or inconsistent.

Material and resource-related conditions also emerged as influential elements of classroom ecology. While supplementary and digital materials expanded instructional possibilities, inconsistent access among students appeared to shape participation patterns. Learners with greater access to supplementary resources engaged more autonomously, whereas those without such access tended to participate more passively or disengage. This observation resonates with van Lier’s (2004) view that participation is shaped by the affordances available to learners within their immediate environment. In the present study, material conditions influenced how students engaged, the forms of participation those were possible, and the perceived relevance of communicative activities.

Finally, a misalignment between curriculum outcomes and classroom assessment practices was evident across participant accounts. Although the curriculum emphasizes balanced development of language skills, assessment practices reported by teachers remained largely oriented toward multiple-choice test performance. Teachers indicated that speaking and writing were not consistently evaluated, partly because opportunities to meaningfully develop these skills were constrained by time and pacing demands. Within the scope of this study, this outcome–practice–assessment tension highlights how communicative intentions articulated in curriculum documents may be moderated by local evaluative priorities. Similar tensions have been documented in recent curriculum and classroom research, which shows that assessment-driven accountability structures and pacing demands often constrain communicative intentions at the classroom level, producing gaps between policy discourse and enacted practice (Priestley et al., 2015; Sato and Loewen, 2022).

Taken together, these findings suggest that the enactment of communicative language curricula is shaped not only by task design or teacher effort, but also by the relational, emotional, material, and evaluative conditions of schooling. From a responsive evaluation perspective, the study demonstrates how curriculum challenges that often remain obscured in outcome-based evaluations can be rendered visible. These include socio-emotional barriers to participation, tensions between pacing demands and communicative goals, and inequities in material access.

Recent classroom ecology and interaction-focused research similarly emphasizes that learners’ willingness to participate and engage communicatively is co-constructed through affective safety, peer dynamics, and material affordances rather than instructional design alone (Woods and Copur-Gencturk, 2024). In this sense, the present findings extend this literature by demonstrating how such interactional and emotional constraints intersect with curriculum enactment and assessment pressures in everyday classroom contexts.

By foregrounding stakeholder experiences, responsive evaluation enables the identification of context-specific constraints that shape curriculum enactment. Such insights can inform adaptive curriculum support, teacher professional development, and material design sensitive to classroom ecology and learner readiness. Addressing misalignments between curriculum intentions and classroom practice may therefore benefit from attention to the socio-emotional climate of participation, pacing and assessment structures, equitable access to learning materials, and sustained support for teachers’ pedagogical mediation within specific educational contexts.

## 6 Conclusion

This study examined the implementation of the 5th-grade English curriculum through a responsive evaluation approach, focusing on the socio-emotional and ecological conditions shaping communicative participation. The findings indicate that the curriculum’s communicative intentions appeared to be mediated, within the studied settings, by performance-oriented institutional expectations, uneven socio-emotional readiness among learners, variability in classroom material conditions, and assessment practices oriented toward measurable outcomes. As a result, the enacted curriculum often differed from the written curriculum, particularly in relation to opportunities for speaking and listening development.

The findings suggest that communicative competence is not fostered solely through the inclusion of tasks in curricular documents, but through classroom environments that support emotional security, meaningful interaction, and learner agency. Teachers in this study actively worked to sustain engagement through pedagogical and emotional mediation; however, these efforts were shaped by pacing pressures, limited task variation in instructional materials, and uneven access to supplementary resources. Enhancing communicative language learning may therefore involve attending not only to instructional practices, but also to the contextual conditions that enable such practices.

Based on the findings, several context-sensitive implications may be considered. Curriculum implementation schedules may benefit from greater flexibility, allowing teachers to allocate time for interactional activities without creating tension with assessment expectations. The development and distribution of instructional materials may also prioritize equitable access and provide richer scaffolding for oral and collaborative communication. In addition, professional development initiatives may support teachers in designing classroom environments that foreground relational and emotional dimensions of participation, particularly during transitional grade levels such as Grade 5.

Future research may explore longitudinal trajectories of learners’ communicative confidence and identity development across the middle school years, as well as the role of peer dynamics and broader school climate in shaping willingness to communicate over time. Such work would further illuminate how socio-emotional readiness and classroom ecology interact across contexts, offering theoretically grounded insights for curriculum design and sustained teacher support.

Overall, this study underscores that communicative language learning is an ecological and relational process. Strengthening alignment between curriculum intentions and classroom realities may benefit from careful attention to the emotional, material, and institutional conditions through which language learning unfolds in specific educational settings.

### 6.1 Implications for practice and policy

The findings suggest that fostering communicative language learning requires attention to classroom ecology and socio-emotional conditions, in addition to curriculum content. Within school settings, more flexible pacing guidelines may allow teachers additional time to scaffold interaction, particularly during transitional grade levels. Similarly, enhancing the quality and accessibility of instructional materials may help address participation disparities associated with unequal access to supplementary resources.

Professional development initiatives may support teachers in designing affectively supportive communicative environments, particularly by emphasizing strategies that reduce performance anxiety and encourage risk-taking in language use. At a broader level, greater coherence between assessment practices and communicative learning goals may contribute to reinforcing the curriculum’s intended emphasis on interaction and experiential language use, while remaining sensitive to local implementation contexts.

### 6.2 Implications for future research

Future research may examine how socio-emotional readiness and communicative participation develop longitudinally across the middle school years. Comparative studies across school contexts with differing resource profiles may further illuminate how material affordances shape classroom interactional ecology. In addition, research exploring peer dynamics, belonging, and identity formation in foreign language learning could deepen understanding of how emotional and relational conditions influence learners’ willingness to communicate over time. Also, future research may complement such qualitative analyses with systematic participation mapping, structured interaction coding, or validated socio-emotional measures such as willingness-to-communicate, anxiety, or belonging scales. Such mixed-method approaches would provide additional triangulation and further strengthen evidence regarding socio-emotional processes in L2 learning contexts.

## Data Availability

Because the dataset includes interviews and focus group data with minors and school-based participants, the full transcripts, audio files, and contextually identifiable materials cannot be shared publicly for ethical and confidentiality reasons. However, de-identified analytic materials (e.g., coded extracts, the coding framework, and documentation of theme development) can be made available upon reasonable request to the corresponding author, subject to ethical approval and confidentiality protections.
